# Generation of Common Marmoset Model Lines of Spinocerebellar Ataxia Type 3

**DOI:** 10.3389/fnins.2020.548002

**Published:** 2020-09-24

**Authors:** Ikuo Tomioka, Yoshitaka Nagai, Kazuhiko Seki

**Affiliations:** ^1^Department of Biomedical Engineering, Shinshu University, Nagano, Japan; ^2^Department of Neurophysiology, National Institute of Neuroscience, National Center of Neurology and Psychiatry, Tokyo, Japan; ^3^Department of Neurotherapeutics, Osaka University, Graduate School of Medicine, Osaka, Japan; ^4^Department of Degenerative Neurological Diseases, National Institute of Neuroscience, National Center of Neurology and Psychiatry, Tokyo, Japan

**Keywords:** spinocerebellar ataxia type 3, transgene, neurodegenerative disease, Tet-inducible gene expression system, ATXN3

## Abstract

Animal models are indispensable tools in the development of innovative treatments for rare and incurable diseases. To date, there is almost no effective treatment for neurodegenerative diseases, and animal models that properly simulate human disease pathologies are eagerly anticipated to identify disease biomarkers and develop therapeutic methods and agents. Among experimental animals, non-human primates are the most suitable animal models for the study of neurodegenerative diseases with human-specific higher brain dysfunction and late-onset and slowly progressing symptoms. With the rapid development of novel therapies such as oligonucleotide therapeutics and genome editing technologies, non-human primate models for neurodegenerative diseases will be essential for preclinical studies and active interventional trials. In a previous publication, we reported the generation of the first transgenic marmoset model of spinocerebellar ataxia type 3 and successful obtainment of subsequent generations with stable disease onset. Moreover, we generated transgenic marmosets in which the transgene was controlled by the tetracycline-inducible gene expression system. In this mini-review, we summarize the research on our marmoset model of spinocerebellar ataxia type 3.

## Introduction

Spinocerebellar ataxia type 3 (SCA3), also called Machado-Joseph disease, is an autosomal, dominantly inherited neurodegenerative disorder that exhibits progressive cerebellar ataxia accompanied by other neurological symptoms, including bulbar dysfunction, pyramidal and extrapyramidal signs, and peripheral nerve impairment (Paulson, 2012). SCA3 is caused by an expansion mutation of cytosine-adenine-guanine (CAG) repeats encoding a polyglutamine stretch from 51 to 91 repeats in the ataxin-3 gene, which normally has fewer than 44 CAG repeats (Kawaguchi et al., 1994; Durr et al., 1996; Padiath et al., 2005; Colomer Gould, 2012; Donis et al., 2016). Novel therapies have been developing rapidly for clinical application, including the suppression of mutant ataxin-3 gene expressions using antisense oligonucleotides (ASOs) (Moore et al., 2017; McLoughlin et al., 2018), the inhibition of polyQ protein aggregation by arginine (Minakawa et al., 2020), and the acceleration of autophagic clearance of expanded polyQ tract (Li et al., 2019). In particular, antisense oligonucleotides (ASOs) represent a non-viral gene suppression approach that has emerged as a compelling therapeutic strategy for neurologic, oncologic, cardiac, and metabolic disorders. However, there is no effective treatment currently available to cure or delay onset of SCA3 patients, like for most other neurodegenerative diseases. To develop therapeutic agents and biomarkers for neurodegenerative diseases, clinically-relevant animal models that recapitulate the human disease pathology are eagerly anticipated.

Animal models of neurodegenerative disorders have been developed mainly in mice to understand the mechanisms of the diseases and identify new therapies and biomarkers. However, differences between humans and rodents in the structure and physiological functions of the brain have resulted in difficulty reproducing the selective vulnerability of specific neurons or circuits in mouse and rat models (Chan, 2013; Tomioka et al., 2018). In addition, the small-sized brains of rodents are difficult to be analyzed anatomically or functionally in detail by *in vivo* imaging techniques such as magnetic resonance imaging (MRI) or positron emission tomography (PET). These limitations have resulted in the failure to predict the efficacy of clinical trials in human patients from the experimental findings obtained from rodent models of neurodegenerative diseases. Non-human primates, on the other hand, share strikingly similar genetic, physiological, and behavioral traits with humans and can provide a better test system for drug and biomarker discovery. They also recapitulate the human aging process; therefore, they are the most appropriate model for late-onset neurodegenerative diseases. Before human clinical trials, the validation of novel treatments and drugs for neurodegenerative diseases in non-human primates is often essential. Additionally, non-human primates are important models for evaluating human-like behavioral alterations or cognitive decline that are difficult to investigate in other animal models. Therefore, they are extremely valuable in improving our knowledge of and establishing new potential therapeutic strategies for neurodegenerative diseases like SCA3.

Despite their value, non-human primates are not widely used due to the limited availability of the animals, who require a large breeding space and specialized care and come with high costs and potential ethical concerns (Nadon, 2006). Among non-human primates, the common marmoset (*Callithrix jacchus*) is a small, non-endangered New World primate that is native to Brazil and is an established model for neuroscience, infectious disease, behavioral research, obesity, and reproductive biology (Mansfield, 2003; Colman, 2018). The common marmoset has the advantage of its small size (about 250–450 g), which makes handling and housing more convenient compared with the macaque (Yun et al., 2015). In captivity, mortality increases from 35 to 85% between 5 and 10 years of age (Tardif et al., 2011). Similar to that of the rhesus monkey, the genome of the common marmoset shares about 93% sequence homology with the human genome (Marmoset Genome and Analysis, 2014), and although marmosets are not as genetically tractable as mice, stable transgenic marmosets capable of transmitting the transgene to their offspring have been generated (Sasaki et al., 2009; Tomioka et al., 2017a, b). Additionally, compared with other laboratory primates, marmosets offer many advantages, such as a shorter gestation period, faster sexual maturation, and higher fecundity, permitting the rapid establishment of gene-modified model lines (Tomioka et al., 2018). They are reproductively competent at ∼1.5 years of age, produce litters of 2–3 offspring every 5–6 months, and are considered aged at 8 years of age (Abbott et al., 2003; Tardif et al., 2003; Colman, 2018). This makes the marmoset a particularly attractive model for neurodegenerative diseases.

## Genetic Modification Techniques for Marmosets

Mammalian genetic modification techniques have always been established first in mice. Although a huge number of genetically modified models have been created since the birth of the first transgenic mouse (Jaenisch and Mintz, 1974), genetic engineering in non-human primates is much more difficult (Chen et al., 2016). For instance, creating transgenic marmosets with germline transmission required 35 years of research since the first transgenic mice were established (Jaenisch and Mintz, 1974; Sasaki et al., 2009). Among several methods for the creation of transgenic animals, lentiviral transgene introduction is currently accepted to generate transgenic marmosets (Sasaki et al., 2009) because of its extremely high efficiency rate of close to 100%. However, lentiviral vectors carry several lingering issues regarding genetic mosaicism, variability of transgene expression, and packaging size. In general, the number of integration sites found in lentiviral vector-produced transgenic animals has ranged between 1 and 31 copies (Park, 2007). Lentiviral vectors are still susceptible to chromosomal position effects that result in transgene silencing or variegated expression. Moreover, transgenes markedly larger than 8 kb have reduced transgenesis rates using lentiviral vectors because of the upper limit of the lentiviral vector packaging capacity. Although further studies are needed to improve these issues, lentiviral-mediated gene transfer seems likely to continue as the main delivery vehicle for creating transgenic marmosets due to the appeal of its extremely high efficiency rate.

## The Transgenic Marmoset Model of SCA3

### Transgenic Marmoset Model of SCA3 Recapitulating Progressive Neurological Symptoms

In 2017, the first transgenic marmoset model of SCA3 was generated using lentiviral transgene introduction based on a construct containing 120 CAG repeats (Tomioka et al., 2017a). Hyperexpansion of the CAG repeat above the human disease range (51–91 repeats) has been widely applied to reproduce neurodegenerative diseases in short-lived rodent models, because the number of CAG repeats is inversely correlated with disease onset in the polyQ diseases (Davies et al., 1997; Watase et al., 2002; Boy et al., 2010). Non-human primates and their gametes are valuable and difficult to collect in sufficient quantity; therefore, we used lentivirus-mediated gene transfer that has hitherto been the most successful method of producing transgenic nonhuman primates (Sasaki et al., 2009; Chan, 2013). Moreover, because triplet repeat sequences are known to show instability during DNA replication (Lenzmeier and Freudenreich, 2003), a total of four CAA triplets that also encode glutamine were introduced every 30 CAG repeats within the full-length ATXN3 cDNA (transcript variant ad, also known as variant 3) to avoid mutation. To ensure the production of transgenic marmoset models that are expected to develop neurologic symptoms at an early age, the transgenes were driven by a cytomegalovirus (CMV) promoter that induces ubiquitous and strong transgene expressions.

**FIGURE 1:**
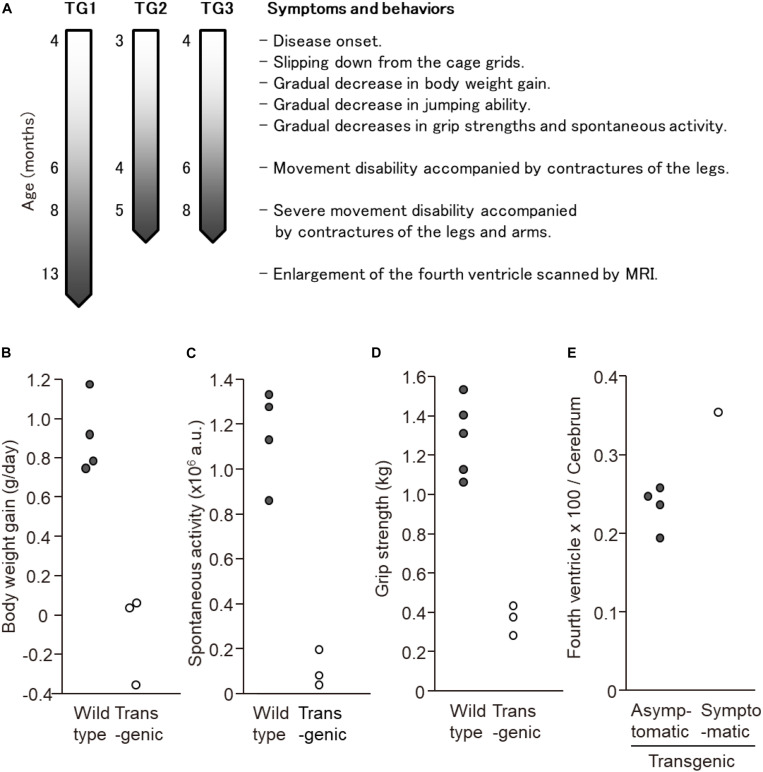
Behavioral analyses of the three symtptomatic transgenic marmosets. **(A)** Schematic of symptom progression in the three symtptomatic transgenic marmosets. **(B)** The levels of body weight gain, **(C)** spontaneous activity level, and **(D)** grip strength of three symptomatic transgenic marmosets were significantly lower than those of wild-type marmosets at 5–6 months of age. **(E)** MRI scanning revealed significant enlargement of the fourth ventricle in the symptomatic transgenic marmoset 1 compared with asymptomatic transgenic marmosets at 13 months of age. **(B–E)** Adapted and summarized from “Transgenic Monkey Model of the Polyglutamine Diseases Recapitulating Progressive Neurological Symptoms” by Tomioka et al. (2017a) with permission.

**FIGURE 2:**
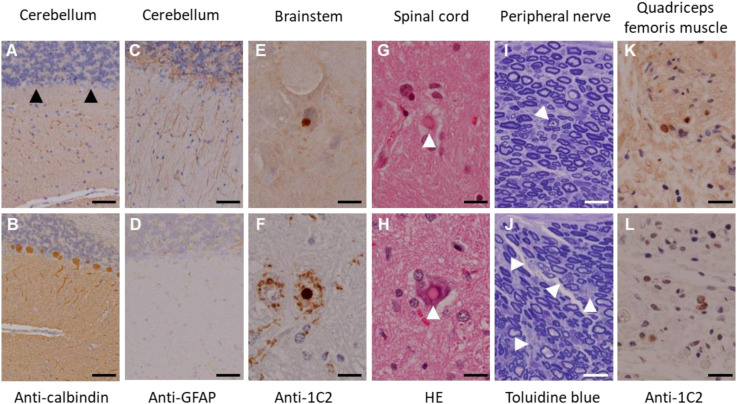
Immunohistochemical analyses of the founder transgenic marmoset 1 (top) and 2 (bottom). **(A,B)** Anti-calbindin staining of the cerebellum. Arrowheads indicate degenerated Purkinje cells. **(C,D)** Anti-GFAP staining of the cerebellum. **(E,F)** Anti-GFAP staining of the brainstem. **(G,H)** Hematoxylin and eosin staining of the spinal cord. Arrowheads indicate inclusion bodies. **(I,J)** Toluidine blue staining of the peripheral nerves of the upper limbs. Arrowheads indicate axonal degeneration. **(K,L)** Anti-1C2 staining of the quadriceps femoris muscle. Scale bars: 100 μm in **(A–J)**; 20 μm in **(K,L)**. **(D,H–L)** Adapted and summarized from “Transgenic Monkey Model of the Polyglutamine Diseases Recapitulating Progressive Neurological Symptoms” by Tomioka et al. (2017a) with permission. The other images in this figure are from the personal bank of images.

In this experiment, a total of seven transgenic marmosets were born from 66 transgene-introduced embryos, this low birth ratio might have been caused by the cytotoxic effects of strong transgene expression induced by the CMV promoter. Although most fetal transgenic marmosets were miscarried in the early gestational period, all offspring showed no neurological symptoms at birth. Among them, three marmosets, transgenic 1 to 3, with higher transgene expression developed neurological symptoms of varying degrees at 3–4 months after birth, followed by gradual decreases in body weight gain, jumping ability, spontaneous activity, and grip strength, indicating time-dependent disease progression (Figure 1A). The levels of body weight gain (Figure 1B), spontaneous activity (Figure 1C), and grip strengths (Figure 1D) in the symptomatic transgenic marmosets were significantly lower than those of wild-type marmosets at 5–6 months of age. MRI scanning revealed significant enlargement of the fourth ventricle in the symptomatic transgenic marmoset 1 (Figure 1E) compared with asymptomatic transgenic marmosets, suggesting cerebellar atrophy. Immunohistochemical analysis was performed at 13.5 months of age for transgenic marmoset 1 and at 5.5 months of age for transgenic marmoset 2. The results of transgenic marmoset 1 revealed a marked loss of Purkinje cells (Figure 2A) accompanied by gliosis (Figure 2C) in the cerebellum, whereas those of transgenic marmoset 2 showed no such pathology (Figures 2B,D). Also, both transgenic marmosets 1 and 2 showed the inclusions in the brainstem (Figure 2E,F), spinal cord (Figures 2G,H), and quadriceps femoris muscle (Figures 2K,L), as well as the axonal degenerations in of the peripheral nerves of the upper limbs (Figures 2I,J). Intriguingly, our marmoset model seemed to exhibit, at least in part, selectivity in the affected tissues despite the widespread expression of mutant ATXN3 protein under the CMV promoter. In human SCA3 patients, endogenous mutant ATXN3 expression is also widespread even though neurodegeneration is selective (Nishiyama et al., 1996). Indeed, involvement of the cerebellum, the anterior horn of the spinal cord, and the peripheral nerves is often observed in SCA3 patients (Paulson, 2012).

Random integration effects of the lentiviral vector, including variations in copy number and integration site, might have resulted in variability in behavioral and pathologic phenotypes among our first-generation transgenic marmosets. To date, we have successfully obtained third-generation transgenic marmosets that carry the transgene with 120 CAG repeats, showing that the transgene is steadily transmitted to offspring. Notably, the second and subsequent generations of transgenic marmosets exhibited disease onset followed by progressive motor impairment after growing. Compared with founder transgenic marmosets, delayed disease onset and mild symptom progression were observed in second and subsequent generations of transgenic marmosets (Figure 1). As the generations progressed, multiple transgene insertions in different chromosomes of founder transgenic marmosets purified into two sites in second and subsequent generations. Considered together with the integration sites and the period of disease onset found in second and subsequent generations of transgenic marmosets, one integration site is estimated to be important for the development of symptoms. Therefore, there is a high possibility of the establishment of a pure and stable disease model line in the next generation.

We believe that our marmoset model with stable disease onset and slow symptom progression will be useful for various studies on neurodegenerative diseases, including the identification of disease biomarkers that could not be achieved with mouse models, and will play a critical role in future therapies for neurodegenerative diseases, as well as the evaluation of the efficacy and metabolic profiles of therapeutic candidates (Morton and Howland, 2013; Howland and Munoz-Sanjuan, 2014). In fact, a human biomarker candidate has been successfully detected using our model marmosets (*unpublished*), and it is highly likely that novel biomarkers discovered in the future will be directly linked to humans. Since our model marmosets expressed the mutant full-length ATXN3 mRNA without 5′- and 3′-UTR region under the CMV promoter, they could be used for preclinical therapeutic trials based on the suppression of mutant *Atxn3* gene expression using antisense oligonucleotides (ASOs) targeting the coding mRNA sequence, the inhibition of polyQ protein aggregation, and the acceleration of autophagic clearance of expanded polyQ tract, but not for trials targeting intrinsic promoter activity, pre-mRNA splicing, and UTR transcription. ASOs targeting exon 3 of ATXN3 mRNA also cannot be used in our marmoset model because the ATXN3 transcript variant ad lacks this region. Although the insertion of CAA triplets into every 30 CAG repeats within the full-length ATXN3 cDNA has the advantage of making a stable SCA3 marmoset model without a repeat mutation, it cannot be used to studies the instability of CAG repeats that occurs between generations as well as studies of RNA’s toxicity. While the introduction of 120 CAG repeats over a range of human SCA3 patients is promising for the creation of an early onset model, we cannot rule out the possibility that the symptoms of the marmoset models may be artifacts due to overextension.

### Transgenic Marmoset Model of SCA3 With Tetracycline (Tet)-Inducible Gene Expression System

Recently, SCA3 has been investigated as a disease targetable by ASO treatment in mouse models (McLoughlin et al., 2018). The identification of efficacious therapies for patients with neurodegenerative diseases has been limited by the great degree of variability in biochemical and clinical features of the diseases, which makes it difficult to assess their response to therapy. Therefore, there is an urgent need for sensitive post-therapeutic biomarkers to evaluate therapeutic efficacy in clinical trials and to monitor the responses of patients to new therapies.

Our previous transgenic marmoset model that transgenes are driven by the CMV promoter showed juvenile disease onset, and rapid disease progression which are unusual in human patients with SCA3. It appears that although ubiquitously expressed promoters induce strong transgene expression, they also lead to clinically irrelevant symptoms. Therefore, in addition to previous SCA3 model marmosets whose transgenes were driven by the CMV promoter, we generated a transgenic marmoset model line of SCA3 in which transgenes were controlled by the Tet-inducible gene expression system (Tomioka et al., 2017b). The ability to control the disease onset is an important advantage in biomarker development studies because it allows us to define the disease onset at the molecular level. Although, apparent symptoms were not observed, those marmosets showed inducible transgene expression with doxycycline treatment for 7 days. The main advantage of the Tet-inducible gene expression system is its ability to turn transgene expression on and off by adding tetracycline or its derivate, doxycycline, thereby enabling us to distinguish phenotypes and molecular biology before and after disease onset. The Tet-inducible gene expression system will also enable us to study disease mechanisms and possibly the prevention of disease progression. Blocking the transgene expression led to the disappearance of inclusions and amelioration of the behavioral phenotype, suggesting that neurodegenerative disease is reversible. This feature is important in analogizing the timing of appropriate therapeutic interventions in human patients. An SCA3 mouse model using this system also revealed that reducing the production of pathogenic ATXN3 may be a promising approach for treating SCA3 (Boy et al., 2009). The fact that phenotypic and pathological features were reversed in a conditional mouse model of SCA3 by switching off the transgene indicates that inhibiting transgene expression after disease onset simulates a post-therapeutic state. Therefore, our Tet-On transgenic marmoset model also could simulate a post-therapeutic state, and it will be a powerful tool for identifying novel biomarkers that are highly translatable to humans.

## Perspectives

To create a non-human primate model that accurately reproduces human disease pathology, it is important to develop advanced genetic modification techniques such as genome editing. Precise and complicated genome modifications, such as targeted gene knock-in and making or repairing point mutations without occurrence of genetic mosaicism, remain impractical in non-human primates, largely due to the low efficiency of these techniques (Park and Silva, 2019). Knockout marmosets produced by genome editing technologies were reported recently (Sato et al., 2016; Kumita et al., 2019), and the application of these technologies has been much faster compared with previous transgene introduction methods. A base editor that enables the direct and irreversible conversion of target DNA base (Komor et al., 2016; Gaudelli et al., 2017) is also an attractive tool for making various marmoset models in the future. This system has proven to be a highly efficient method for generation of targeted point mutations even in human embryos (Li et al., 2017). Although the development of the advanced genetic modification techniques described above is an important issue for the future, faithfully reproducing the characteristics of late-onset will result in difficulties in neurodegenerative disease research. Therefore, it is necessary to apply not only genome editing techniques but also conventional gene modification techniques developed in mice to non-human primates to develop various models suitable for each study. Besides, marmosets offer several advantages such as reduced intergeneration time which allows the establishment of transgenic lines two-three times faster than in macaques (Park and Silva, 2019), a transgenic marmoset model will be a powerful tool for the preclinical study of neurodegenerative diseases, and continuous efforts for the development of efficient genetic tools adapted to them will be important.

Now, novel findings are being discovered that cannot be obtained with any model animals other than non-human primates, and we are confident that those findings from our marmoset model can contribute to overcoming neurodegenerative diseases. Although non-human primates are highly valued in biomedical research because of their genetic similarity to humans, their similarity to humans also raises specific ethical concerns about their use in scientific experiments. All our animal experiments were conducted in accordance with institutional guidelines and the National Research Council’s Guide for the Care and Use of Laboratory Animals. We adhere to the 3R principles, and the application of the 3R principle to the use of marmoset can help to improve the quality of life of our marmoset models. We also declare that we will continue to develop better research protocols and higher standards for captive management, which could result in improvements in data quality, and the reliability of some research results.

## Author Contributions

IT, YN, and KS developed the theoretical framework. IT and YN wrote the manuscript. All authors contributed to the article and approved the submitted version.

## Conflict of Interest

The authors declare that the research was conducted in the absence of any commercial or financial relationships that could be construed as a potential conflict of interest.

## Abbreviations

ASO: antisense oligonucleotide
ATXN3: ataxin 3
CMV: cytomegalovirus
SCA3: spinocerebellar ataxia 3
Tet: tetracycline.
